# Medical Students Perceive Better Group Learning Processes when Large Classes Are Made to Seem Small

**DOI:** 10.1371/journal.pone.0093328

**Published:** 2014-04-15

**Authors:** Juliette Hommes, Onyebuchi A. Arah, Willem de Grave, Lambert W. T. Schuwirth, Albert J. J. A. Scherpbier, Gerard M. J. Bos

**Affiliations:** 1 Department of Educational Development and Research, Faculty of Health Medicine and Life Sciences, Maastricht University, Maastricht, The Netherlands; 2 Department of Epidemiology, The Fielding School of Public Health, University of California Los Angeles, Los Angeles, California, United States of America; 3 Flinders Innovation and Clinical Education, Flinders Medical School, Flinders University, Adelaide, Australia; 4 University Hospital Maastricht, Department of Internal medicine, Haematology, Maastricht, The Netherlands; University of Minho, Portugal

## Abstract

**Objective:**

Medical schools struggle with large classes, which might interfere with the effectiveness of learning within small groups due to students being unfamiliar to fellow students. The aim of this study was to assess the effects of making a large class *seem* small on the students' collaborative learning processes.

**Design:**

A randomised controlled intervention study was undertaken to make a large class seem small, without the need to reduce the number of students enrolling in the medical programme. The class was divided into subsets: two small subsets (n = 50) as the intervention groups; a control group (n = 102) was mixed with the remaining students (the non-randomised group n∼100) to create one large subset.

**Setting:**

The undergraduate curriculum of the Maastricht Medical School, applying the Problem-Based Learning principles. In this learning context, students learn mainly in tutorial groups, composed randomly from a large class every 6–10 weeks.

**Intervention:**

The formal group learning activities were organised within the subsets. Students from the intervention groups met frequently within the formal groups, in contrast to the students from the large subset who hardly enrolled with the same students in formal activities.

**Main Outcome Measures:**

Three outcome measures assessed students' group learning processes over time: learning within formally organised small groups, learning with other students in the informal context and perceptions of the intervention.

**Results:**

Formal group learning processes were perceived more positive in the intervention groups from the second study year on, with a mean increase of β = 0.48. Informal group learning activities occurred almost exclusively within the subsets as defined by the intervention from the first week involved in the medical curriculum (E-I indexes>−0.69). Interviews tapped mainly positive effects and negligible negative side effects of the intervention.

**Conclusion:**

Better group learning processes can be achieved in large medical schools by making large classes seem small.

## Introduction

Powerful learning environments comply with the cognitive architecture of learning [1] combining learning within a meaningful context (contextualism), learning as an active process (constructivism) and learning in groups (collaboration). These learning environments assemble small groups as the units in which learning takes place to ‘teach’ undergraduate medical students. In such small groups, students are supposed to solve meaningful problems, share information and discuss conflicting ideas [2]. These distinctive steps in the process of learning have shown positive effects on short- and long-term knowledge acquisition [3]–[6]. Performing these steps within a small group has been shown more effective than acted by an individual alone [7], [8]. Furthermore, in the field of medical education positive effects of these group processes on a variety of medical competencies have been shown repeatedly [9]–[12]. In accordance with the evidence, many medical schools all over the world have changed their learning context towards powerful learning environments.

Medical schools have grown towards ‘mega’ classes with commonly over 300 students in European medical schools. This scale enlargement has shown to make teaching large classes difficult [13]–[15]. In these traditional learning contexts the solution to involve all students in the mega-class was to include group-based activities in the learning contexts [16]. However, in powerful learning contexts, which are founded upon learning in small groups, it is plausible that the scale enlargement threatens the students' learning processes. Random allocation of students into new groups every few weeks again results in small groups of unfamiliar students. Sharing knowledge in the collaborative process incurs an implicit cost, while the expected returns of relevant new knowledge and/or expertise are uncertain. Some students are therefore less willing to share knowledge than others. Group member familiarity might reduce costs of sharing information among students [17], [18]. Second, groups must invest time and energy in the collaborative process before the group can become effective [19]. Changing the composition of groups too quickly might prevent groups from reaching the beneficial effects of collaboration in groups. Such suboptimal effectiveness of physicians' medical training increases the need for parsimonious solutions to make medical education more effective.

**Figure 1:**
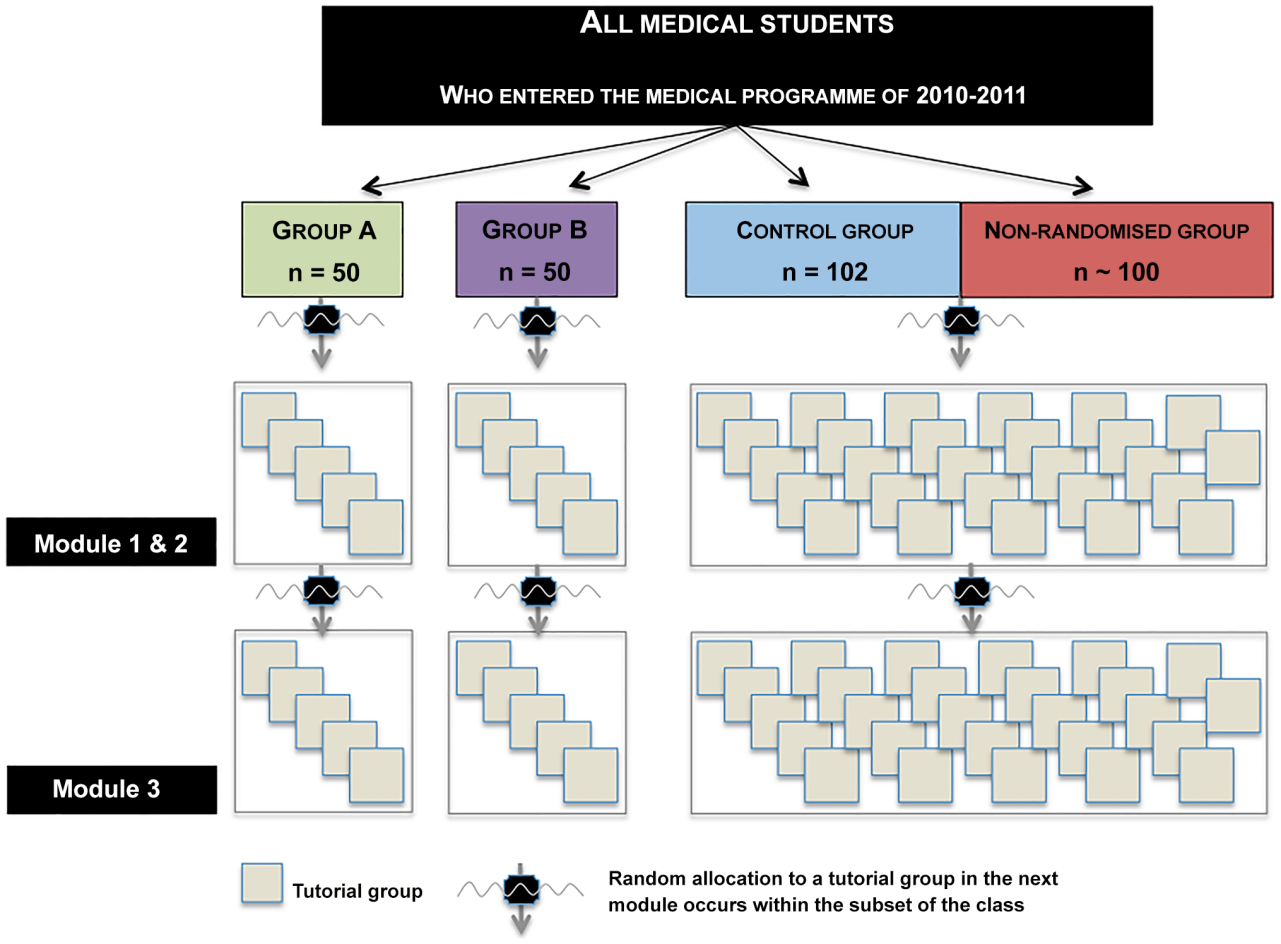
Overview of the randomised controlled trial allocation procedure and intervention. The class of 2010–2011 was randomised into two intervention groups (A and B) with 50 students in each subset and the control *group (n = 102). The control group was mixed with the non-randomised students representing the large subset of the class. The intervention consisted of allocating students within the subsets of the class to new tutorial groups while progressing through the curriculum. This way, students in the small subsets frequently interacted with the same students over time.*

The battle against scale enlargement cannot be won by reducing the number of students enrolling in medical schools. Dividing a medical parallel programme into two (or more) sections will result in a duplication of necessary staff time [20]. Next, in medical education we strive towards evidenced-based decision making to design the most powerful learning context. Therefore, our study angled this large class debate differently. We conducted a randomised controlled intervention study to test if subdividing a large class into small subsets could facilitate the students' collaborative learning processes positively. This division of a large class in small subsets, which is schematically illustrated in Figure 1, makes students collaborate with the same students frequently in small groups. Hereby, these subsets increase the time students spend collaborating with the same students they have collaborated with in the past. More time to collaborate with one another aligns with the group development literature [19], [21] which has shown to increase the effectiveness of groups.

Our primary aim was to assess group dynamics in small groups in the medical curriculum. As was briefly expounded in the first paragraph, interaction among students when problem solving, sharing knowledge and discussing medical scenarios are the steps in which learning takes place. However, learners in groups do not necessarily interact when present in the same space at the same time [22], [23]. Attitudes, motivation and beliefs drive or inhibit interaction within a group. For example, potency beliefs -beliefs that the group will be valuable for one's learning process- is an important predictor for effective learning in a group [24], [25]. A strong body of evidence shows that effective learning in groups is dependent on the quality of interaction and a variety of attitudes, motivations and beliefs among the group members [26], [27]. Therefore, we borrowed the parameters of effective group processes as the primary outcome measures: group learning behaviour, potency, social cohesion and psychological safety e.g. [19], [28]. In this study we hypothesized that the students in small subsets perceived better group learning processes, reflected as more positive primary outcome measures compared to the control group.

The second aim of this study, focuses on the informal side of group learning. Students spend the majority of time studying and interacting with other students outside the formally organised activities. Learning in the ‘informal’ context with other students has been found an important part of the students' learning process [29] and could thus confound our findings related to learning in the formally organised groups [30]. Large classes allow students to learn with or from a large number of students. However, large learning networks have been shown to be less effective as it demands much energy to maintain the learning network [31]. Therefore our second hypothesis tested whether dividing the class into subsets directed informal group learning networks towards learning primarily within their own subset.

Finally, to be thorough and not miss (unexpected) effects of this intervention study on the students' group learning process in an actual medical school, the third aim was to explore students' perceptions on the intervention.

## Methods

Research in medical education is exempted from the Medical Ethical Committee in The Netherlands on the ground that this type of research does not intend to answer a research question on health or pathology (aetiology, pathogenesis, symptoms, diagnosis, prevention, results of a treatment) [32]. As the medical educational domain does not agree with this view, a review board for medical education was designed, but was not yet in function when this research was planned and applied. This did not prevent our research team to apply ethical guidelines for our research. To meet the need for thorough ethical guidelines normally tested in an ethical committee, the students were given the details of the intervention, after which they were asked for informed consent to take part. Students could withdraw at any moment without having to provide a reason. The intervention did not influence the contents of the educational programme at all. For each study (observation), students were again briefed with the goals of the research and asked to participate and provide informed consent to use their data for research purposes. Stop criteria were formulated on the basis of achievement, the pre-requisite for students to progress through the medical programme. To replace an independent institutional review board the management team of the medical faculty responsible for the quality of education in the medical programme, approved our research proposal and annual feedback on the progress was given.

### Setting & participants

This study was conducted in the first two years of the undergraduate-entry pre-clinical curriculum in the Maastricht University medical school. This medical school employs a Problem-Based Learning (PBL) approach with small-group tutorials as the backbone of the curriculum in conjunction with practical learning sessions, e.g. gross anatomy laboratory sessions, to complement these groups. The formal activities compose approximately ten hours per week, which leaves plenty of time for informal learning. At its inception in 1974, classes consisted of 34 students. Nowadays, this school enrols approximately 320 students yearly.

Students enrol in a class and participate in a set of modules in chronological order. Recruitment for this study started with enrolment for the new academic year of 2010–2011 (July 2010). One inclusion criterion was used: informed consent to participate in this RCT.

### Randomisation and intervention

Of the students enrolling in the undergraduate-entry Maastricht University medical school, 202 students were randomised into three groups: Two intervention groups (A & B) with 50 students each and the control group (C) consisting of 102 students. For logistical reasons, mainly on the national level, approximately 100 students were not allocated to our medical school before the randomisation of students to tutorial group, which occurs two weeks before the start of the medical programme. Therefore, these students could not participate in the study. The majority of these students still started within the first week of the medical programme. Only eighteen students entered late in the first module. Together with 27 students who did not want to participate in the intervention study, these ‘non-randomised’ students were mixed with the control group, to generate a large subset of the class.

Students were stratified on their Grade Point Average (GPA) as a proxy to divide weak and strong students evenly over all subsets [33], [34]. It was decided to have two intervention groups so that each intervention group could serve as a cross validation for the results of the other group. Randomisation was performed using block randomisation in STATA version 11 [35]. As is shown in Table 1, gender and age did not differ significantly between the intervention groups and the control group. The non-randomised students were slightly but significantly older and had a lower GPA than the control group.

**Table 1:** Characteristics of the students in the subsets.

	Intervention group A	Intervention group B	Control group	Non randomised group
**Number of students**	n = 50	n = 50	n = 102	n∼100
**GPA (1–5)**	2.8	2.9	2.9	2.4 *
**Gender (% female)**	64.0	70.0	64.7	57.5
**Age**	19.0	19.3	19.3	20 **
**Loss to follow up**	8	4	15	n/a
**Stop participation**	2	1	1	n/a

202 students *were randomised in three groups using stratification on the Grade Point Average (GPA). Students in the non-randomised group differed from the control group in the GPA and age respectively.*

*β = −0.53 SE = 0.17 p = 0.002.

**β = 0.68 SE = 0.20 p = 0.001.

*n/a signifies ‘not applicable’. Re-takers of modules were automatically placed in the non-randomised group of students. Therefore, the number of this non-randomised group changed continuously and loss-to-follow-up could not be calculated. Moreover, students that stopped participating in the intervention or control groups were allocated to the non-randomised group of students.*

The intervention consisted of allocating students to small groups within the subsets of the class for two years (as illustrated in Figure 1). As such, in the first curriculum year, time wise 54.7% of all formally organised educational events in small groups (clinical skills training sessions and tutorial groups) were organised within the subsets of the class. In the second curriculum year, students were only allocated into the tutorial groups within the subsets of the class, resulting in 39.4% of all formally organised activities was spend within the subsets of the class. To control costs no extra staff time was used in this intervention study. To strengthen students' awareness of being involved in the intervention, three two-hour workshops were organised in which students were actively involved in icebreaker games.

### Instruments

#### Group processes in the formal learning context

**Figure 2:**
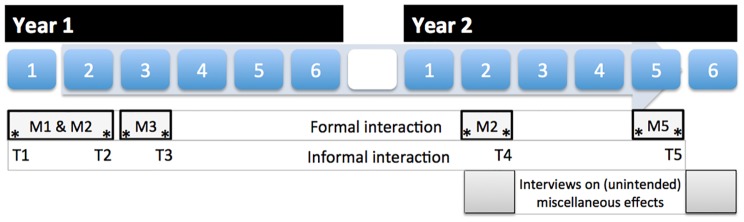
Overview of the instrument assessment over time. Students progressed through six modules every study year. Formal interaction was assessed in the first tutorial group (M1 and M2; since the composition of first two modules did not change), the second tutorial group (M3) and in year two the second and penultimate tutorial groups (M2 and M5 in curriculum year two). The assessment consisted of two observations within the module, indicated as * in the orange boxes. The first observation took place in the second week of the tutorial group and the second in the penultimate week. Informal learning in social networks was assessed during the first three modules in the first year and during two modules in the second study year (T1–T5). Finally, semi-structured interviews assessed the perceptions of the intervention during M2 and M6 of the second curriculum year.

A repeated measures study assessed the primary outcome measure: interaction or collaboration among students in the formally organised small groups over time. The Team Learning Beliefs and Behaviour Questionnaire [28] based on validated scales [36]–[42] assessed learning processes in these formally organised groups by measuring four parameters of effective group processes [43]: group learning behaviour, feelings of psychological safety, social cohesion (communal attraction to the group and its members), and group potency (the belief that the group is effective). These group learning processes were measured on a seven-point scale ranging from (1) ‘I do not agree at all’ to (7) ‘I fully agree’. To assess the developmental aspect of these processes, a longitudinal repeated measures analysis was performed. Figure 2 shows the administration moments over four modules in the two study years. Students were assessed twice per module: in the second week (observation null) and in the penultimate week (observation one).

#### Group processes in the informal learning context

The secondary aim was to study informal group learning among the students, which was quantified by longitudinal social network analyses. Social networks define student learning as interaction between a set of actors or individuals (“nodes”) and their interrelationships (“ties”). Three network types assessed social interaction between students; friendships, giving and receiving information related to the module in which students were involved. Friendship networks explore passive information diffusion, while communication networks have a more instrumental nature (e.g. asking explicitly for help on a certain topic) [44], [45]. Tie strength, the *value of the information that was given or received* respectively, or the intensity of the friendship was measured on a Likert scale ranging from ‘not valuable’ (1) to ‘very valuable’ (5). A previous study demonstrated validity of this method [29]. Five times during the two curriculum years (T1–T5), students' were asked to indicate with whom they interacted in the informal context (outside all formally organised activities), see Figure 2.

#### Perceptions of the intervention

Individual interviews were held with students to explore any (other) positive or negative effects of the randomised controlled trial. Two independent trained interviewers conducted n = 39 and n = 36 interviews with students from the intervention groups A and B and the control group in the beginning and in the end of the second study year (see Figure 2). Students were asked what they noticed from being involved in a small or large subset.

### Analyses

#### Group processes in the formal learning context

Per-protocol multilevel cross-classified regression modelling was used to analyse the data, since two observations per students were obtained while students were involved in tutorial groups, which were assembled from the subsets of the class [46]. The intervention groups (A and B) were compared to the control group. In the large subset of the class, the control group was mixed with non-randomised students. Furthermore, the control group and the non-randomised group of students were compared to ensure that the control group was not put in disadvantage. Response rates varied between 87.6% and 96.6%.

#### Group processes in the informal learning context

Response rates varied between 82.2% and 94.0%. Missing data have considerable negative effects on social network analysis since interpretations of social network relations rely heavily on the assumption that the presence or absence of ties is identified. We dealt with the missing relational data as follows [47], treating the missing ties on the precise estimates of mutuality and other (full) network characteristics to fit from the observed data.

Analyses of social networks started with graphical analysis using Pajek v4G. In order to determine if informal group learning occurred within the subsets of the class, Krackhardt and Stern's External – Internal index was used in UCINET (v6.439) [48]. The E-I index takes the number of ties to members of other subsets of the group (E), subtracts the number of ties to members within the same subset of the class (I), and divides it by the total number of ties in the network. The resulting index ranges from −1 (all ties are only within the subset) to +1 (all ties are outside the subset of the class).

#### Perceptions of the intervention

Thematic analyses [49] was applied to categorise students perception about positive and negative effects of the intervention. The first author (JH) analysed all transcripts. WdG randomly analysed four transcripts to limit reliance of a single researcher. Comparison showed a high level of agreement. Differences were solved by consensus following qualitative research practice [50].

## Results

### Demographics at baseline

Of all randomised students 65.8% were female and the mean age was 19.2 years at the start of the medical course. Four students chose to discontinue participation in the intervention or control group, during the two academic years. Reasons were: planning problems with clinical skills training sessions (n = 2), objections to having exam results analysed (n = 1) or personal dislike of some students within the subgroup (n = 1). Twenty-seven students were lost to follow-up as can be seen in Table 1.

### Group processes in the formal learning context

The intervention was expected to take effect when the small groups were randomised to new small groups at least twice. However, to understand and monitor the effects of mixing the control group and the non-randomised group to generate a large subset, we explored what happened in the first two small groups that students were involved in. In Table 2 and the Table S1 can be found that no significant differences were found between the control group and the non-randomised group.

**Table 2:** Effect sizes of the learning processes in formal groups over time.

	GLB	Potency	Cohesion	Safety
**Year 2 - Module 2**				
**Obs 0** (C)	5.00 (.11)	*4.65 (.12)*	*4.75 (.12)*	*5.10 (.11)*
A	0.42 (.24)	*0.58 (.27)	*0.78 (.24)	*0.47 (.21)
B	0.32 (.24)	0.42 (.26)	*0.80 (.23)	*0.58 (.21)
nR	0.00 (.11)	0.04 (.11)	−0.02 (.12)	0.13 (.12)
**SLOPE** (C)	0.31 (.09)*	0.44 (.10)*	0.55 (.10)*	0.45 (.10)*
A	* −0.39 (.15)	*−0.59 (.17)	*−0.46 (.18)	*−0.39 (.17)
B	−0.27 (.14)	*−0.48 (.16)	*−0.59 (.17)	*−0.52 (.16)
nR	−0.17 (.12)	−0.19 (.13)	−0.11 (.14)	*−0.28 (.13)
**Year 2 - Module 5**				
**Obs 0** (C)	4.93 (.08)	*4.66 (.08)*	*4.63 (.09)*	*5.08 (.08)*
A	0.19 (.14)	*0.28 (.13)	*0.77 (.19)	*0.37 (.16)
B	* 0.28 (.14)	*0.27 (.13)	*0.73 (.18)	*0.36 (.15)
nR	*−0.20 (.10)	−0.06 (.10)	−0.12 (.11)	−0.06 (.11)
**SLOPE** (C)	0.29 (.07)*	0.32 (.07)*	0.41 (.08)*	0.29 (.08)*
A	0.10 (.12)	0.05 (.13)	*−0.27 (.15)	0.02 (.14)
B	−0.10 (.12)	−0.20 (.13)	*−0.36 (.14)	−0.11 (.13)
nR	0.18 (.10)	0.11 (.10)	0.06 (.12)	0.09 (.11)

*Hierarchical cross-classified data analyses reveal that the intervention groups A and B perceive higher group learning processes in curriculum year 2 compared to the control group (C) at observation 0, the start of the module. GLB: Group learning behaviour, Potency: Group Potency, Cohesion: Social cohesion, Safety: Psychological Safety. Effect sizes are given in regression coefficients, with standard errors between brackets. Obs 0: starting point in the module. Slope: increase (β) between the start and the end of the module. C = control group, A & B are the intervention groups (small subsets), and nR is the non-randomised group of students.*

*signifies p-value≤0.05.

**Figure 3:**
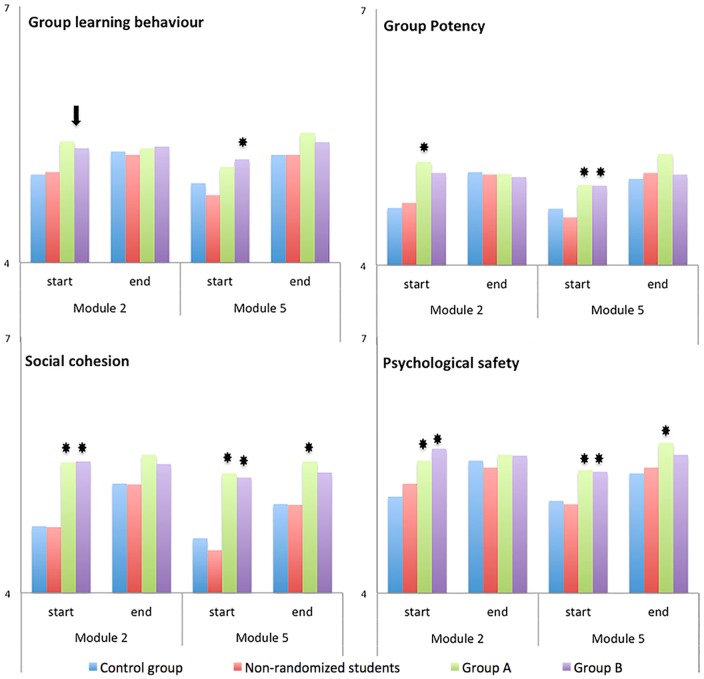
Learning processes in formal groups. This Figure depicts mean perceptions of the four parameters for effective group processes. These learning processes were assessed in two modules in the second curriculum year. The * represents a difference from the control group with a p-value≤0.05. The arrow represents a significant difference when both intervention groups were combined to improve power and overcome the low number of students in the intervention groups in year 2 (approximately 40 students). Please note that Y-axis starts at 4 since this was ‘neutral’ on the scale.

In the second curriculum year students in the intervention groups generally reported a higher quality of group dynamics at the start of the module than the control group (cf. Figure 3 and Figures S1), confirming our expectations. Effect sizes are shown in Tables 2, 3 and in the Table S1. For example, in the case of psychological safety, students in the intervention groups perceived higher degrees of safety than the control group in Module 2 (M2); [Group A] β = 0.47 (0.21) p<0.001, [Group B] β = 0.58 (0.21) p<0.001. Similarly, in Module 5 (M5), students in the intervention groups reported a higher degree of safety [Group A]: β = 0.37 (0.16) p<0.001, [Group B] β = 0.36 (0.15) p<0.001. As for social cohesion, it is clear that students in the intervention groups reported significantly higher cohesion towards the members of the tutorial group at the start and the end of both modules in year two. Potency seems to be perceived higher in the intervention groups since a significant difference was found in group A (β = 0.58 (0.27)), but only a trend was seen in group B (β 0.42,(0.26)). The scale group learning behaviour based on (co)construction and cognitive conflict in the group, reaches a trend to be perceived higher in both intervention groups but didn't reach significance (as can be seen in Table 2). When controlling for the lack of power as the subset size decreased to approximately 40 students per interventions group in the second curriculum year, these non-significant differences by combining both intervention groups (Table 3), group learning behaviour and potency are perceived significantly higher in the beginning of the module than the control group.

**Table 3:** Learning in formal groups over time: a problem of power?

	GLB	Potency	Cohesion	Safety
**Year 2 - Module 2**				
**obs 0** (C)	*5.00 (.11)*	*4.65 (.12)*	*4.75 (.11)*	*5.10 (.11)*
A+B	*0.37 (.18)	*0.50 (.20)	*0.79 (.18)	*0.53 (.16)
nR	0.00 (.11)	0.04 (.11)	−0.02 (.12)	0.13 (.12)
**Slope** (C)	*0.31 (.09)* *	*0.44 (.10)* *	*0.55 (.10)* *	*0.45 (.010)* *
A+B	*−0.32 (.12)	*−0.53 (.14)	*−0.53 (.14)	*−0.46 (.13)
nR	−0.16 (.12)	−0.19 (.13)	−0.11 (.14)	*0.28 (.13)
**Year 2 - Module 5**				
**obs 0** (C)	*4.93 (.07)*	*4.66 (.07)*	*4.63 (.09)*	*5.08 (.08)*
A+B	*0.24 (.11)	*0.27 (.11)	*0.75 (.14)	*0.36 (.12)
nR	−0.20 (.10)	−0.07 (.10)	−0.12 (.11)	−0.06 (.11)
**Slope** (C)	*0.29 (.07)* *	*0.32 (.07)* *	*0.41 (.08)* *	*0.29 (.08)* *
A+B	−0.00 (.09)	−0.08 (.10)	*−0.32 (.12)	−0.05 (.11)
nR	0.18 (.09)	0.12 (.10)	0.07 (.12)	0.09 (.11)

*Since the subsets of the class (A and B) are composed of only approximately 40 students in curriculum year 2, the lack of power could explain why differences between the control group and the subsets of the classes did not reach significance in year 2. Therefore, the intervention groups were combined in the analyses of the modules in the second year. Again, the control group (C) is compared to the intervention groups (A+B) and the non-randomised student group (nR).*

*signifies p-value≤0.05.

The control group was mixed with non-randomised students and could thus be influenced by the latter group of students. Therefore, it is important to note that in all periods, students in the control group did not differ significantly from students in the non-randomised groups. For all results, including the observations in the first year of the medical programme, we refer to the Table S1.

### Group processes in the informal learning context

**Figure 4:**
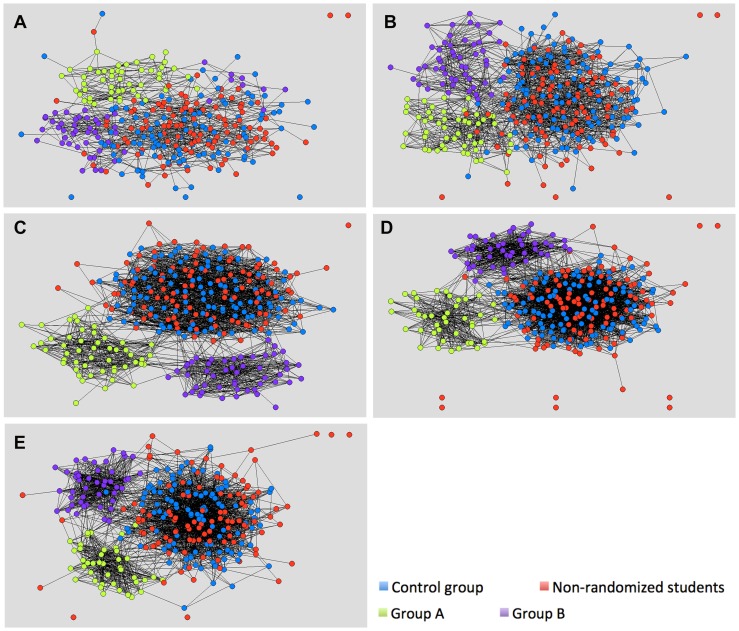
Visualisation of learning among students in the informal learning context over time. *These Figures illustrate how students' learning in the informal context is arranged within the subsets of the class over time (T1–T5). The nodes (students) are connected by lines, which represent information flow among the students. In these learning networks, the lines indicated that student received information from the other students (GET network). The colour of each node depicts the subset of the class.* A = T1, B = T2, C = T3, D = T4, E = T5.

Graphical illustrations represented in Figure 4 and the film S1 with 3D images of the learning networks show that students' build informal social networks within the subsets of the class in either one of the two intervention groups or the large subset over two years.

The E-I indexes (Table 4) quantify the invisible barriers between the subsets showing that the three subsets hardly share any information external to their own subset of the class. These results demonstrate that a simple change in educational design has strong effects on the students' learning process for (at least) 22 months.

**Table 4:** Students learn primarily within the subsets of the class in the informal context.

		T1	T2	T3	T4	T5
**Friendships**	Large subset	−0.90	−0.93	−0.96	−0.95	−0.93
	Group A	−0.75	−0.83	−0.89	−0.81	−0.81
	Group B	−0.69	−0.75	−0.86	−0.86	−0.81
**Giving** module related **information**	Large subset	−0.90	−0.93	−0.96	−0.95	−0.92
	Group A	−0.75	−0.81	−0.85	−0.80	−0.78
	Group B	−0.69	−0.78	−0.85	−0.82	−0.80
**Getting** module related **information**	Large subset	−0.90	−0.93	−0.96	−0.95	−0.92
	Group A	−0.78	−0.81	−0.85	−0.80	−0.78
	Group B	−0.71	−0.78	−0.85	−0.83	−0.81

*The E-I indexes show strong internal orientations when interacting in the informal context over time in three networks (friendship, giving and getting module-related information). The large subset is composed of a mixed group of students from the control group and the non-randomised group. All E-I indexes are significantly different (p<0.05) from H_0_ hypothesis E = I.*

### Perceptions of the intervention

The students in the control group mentioned not to perceive any positive nor negative effects of the randomised controlled trial. The majority of the interviewed students from the intervention groups perceived positive influences from participating in a small intervention group. The positive influences are summarised in Table 5. Students mentioned that they were more familiar to one another, which made collaboration easier. Students were more at ease to ask one another questions and provide feedback to one another. Also, member familiarity made it easier to know what to expect from the other group members. Groups developed rather quickly towards an ‘effective group’. Besides these positive experiences on the process of group learning, students developed a close group of friends and felt rather at home at the university. Finally, students mentioned that they valued to be able to see the personal growth of other students as they met frequently again in tutorial groups over time.

**Table 5:** How 38 students from the intervention groups perceived the intervention.

Advantages	n	%
*I was familiar with the members of tutorial groups which made the group develop more quickly within the modules (from the end of year one and onwards)*	13	34.2
*I was familiar with the members of the tutorial groups, providing me with the feeling that I knew what to expect from the group (members)*	13	34.2
*I was familiar with the members of the tutorial groups, which made collaboration in the group easier*	24	63.2
*I felt that in the tutorial group we were familiar with one another which facilitated providing and receiving feedback to each other*	8	21.1
*I was familiar with the members in the skills training sessions which made me feel more comfortable practising skills on one another*	8	21.1
*I spent much time with students involved in the intervention group which resulted in a close group of friends; this made me feel ‘at home’ in the university*	5	13.2
*We met regularly again in tutorial groups which enabled us to see one another's development in collaboration competencies*	3	7.9

Disadvantages		
*There was a limited number of options to plan skills training sessions*	6	15.8
*I am not familiar to the remaining students of the class*	13	34.2
*If I did not like collaborating with fellow student in the intervention group, there is a high risk to be involved in a future group or assignment again*	3	7.9
*I like meeting new persons every module again, which was not possible in the (small) intervention group*	5	13.2
*Sometimes I expected ‘bad’ collaboration among the members which demotivated me to participate in the group*	2	5.3

Negative influences of the intervention experienced by the 38 students interviewed from the intervention groups (subsets A & B) were limited to uneasy feelings towards collaboration in future groups in which the majority of students will not be familiar to them, not liking to be more frequently involved with a student whom he or she did not like, and fewer time slots to plan clinical skills training sessions (see Table 5).

## Discussion

This randomised controlled study aimed at improving the students' group learning processes in a large class (n∼320). The first outcome measure shows that making a class seem small resulted in more effective group dynamics in formally organised small groups. In other words, changing the composition of small tutorial groups in the medical programme to ensure higher member familiarity among students induced more effective group dynamics as indicated by significantly more positive feelings of psychological safety, social cohesion, group potency and group learning behaviour when a new group was assembled. These findings align with those from the team-based literature describing that group members need to get to know one another before the group can be considered ‘effective’ [19], [43]. Interestingly when comparing effect sizes among the parameters of group dynamics, social cohesion and psychological safety stand out. The literature refers to these perceptions or beliefs as the basis of collaboration in teams [36], [51], [52]. Future studies might be able to determine more specifically how these processes relate to one another to further enhance insights how design features can influence group dynamics. For example, van den Bossche et al [28] suggest that social cohesion and psychological safety are the first steps towards group learning behaviour. However, group potency towards as might grow concurrently with social cohesion *(I like the group members and I belief that as a group we will be able to gain in-depth knowledge into the subject matter)*, and even more strongly after a group had a deep discussion in one of the group sessions. Thus, group dynamics might be related in a non-sequential and more complex cyclical pattern [21], [53]. Although more studies might enable us more insights in the complex matter of group learning behaviour over time, this study does show that a change in curriculum design has a substantial effect on formal group processes. In this study the group processes among the students in the large subset were ‘lower’ as those in the intervention groups for at least 20% of all tutorial group sessions in these two modules. Although this study did not assess group processes during all tutorial groups sessions over all modules, this intervention does indicate that making classes seem small, increases formal group learning for a substantial duration of the formal medical programme.

This study also discovered that the educational design directed informal group learning to occur mainly within the subset of the class over 22 months which might even enhance the effects of the intervention as students spend more time with the same students. At first glance it might seem a disadvantage to be involved in a small subset as this reduces the chance to meet a large number of students. However, in the organisational sciences research has shown that large networks need quite some energy to be maintained [31]. Therefore big networks are not necessarily effective networks. In contrast, safety and judgements of expertise are the strongest indicators of successful informal learning from peers [31]. Since the students in the small subsets indicated to know one another better (interview data) and feel safer among others (formal group learning processes), it is likely that informal learning in networks is also more effective in these small classes. Alternatively, positive experiences during informal group learning networks could contribute to group learning processes in the formally organised small groups. Therefore, this study might have created a better context to learn outside formal activities as well. As students arrange and develop their informal group learning networks themselves, it has been regarded as difficult to steer this learning process in a variety of disciplines [54]. This study is a promising example that it is possible for educators to direct informal learning among students besides formal group learning.

The results of the interviews exploring students' perception of the effects of the intervention support the positive perceptions of students being involved in small subsets. These interviews showed that students perceived higher member familiarity, which in turn improved collaboration among students within the tutorial groups. Moreover, these interviews might indicate that students feel more related to the university when students have developed towards a tight group of friends among close fellow students. Tinto [55] has shown that the curriculum design can make students feel more at home at the university which reduced attrition rates. Future studies might be able to indicate whether subdividing a large class into small subsets indeed causes students to be related closer to the university and results in lower attrition rates as has been shown in the past. Interestingly, students mentioned that it was easier to understand the competency development of fellow students. This intervention might thus be a rather valuable tool for students to receiver better feedback from fellow students, which is in the end a vital method to improving medical competencies of our future medics [56], especially since students tend to be dissatisfied with the feedback they receive [57]. In the end, these interviews show minor to negligible ‘side effects’. What can be learned from these ‘negative’ side effects that can be taken into account when implementing this study into practice, is the ability for students to manage their own agenda. In PBL curricula, students usually have choices in when they can attend skills or anatomy training sessions. These sessions per subset should be flexible enough to fit students' training. We believe that once students choose to be involved in a training session, students also engage more easily in the training session. The literature continues to show that student engagement enhances student learning [58].

Time plays a key role in developing students' collaborative learning processes. We measured processes representing formal group interaction in five modules over two years. Yet, we cannot define precisely the timeframe needed for students in the intervention arm to start experiencing more positive learning processes than the control group. At the same time, within the modules, it cannot be defined when the control group develops to the same level as the intervention arm. This shows that time is still an unsolved parameter with respect to the students' learning process. Previous studies in team learning recognise groups as dynamic social systems changing over time [43]. Future research in education should focus on the influence of time on groups within and over modules.

In the search for evidence-based practice in medical education, randomised controlled trials are valuable tools to show how education can be improved. Especially since very little studies have applied this research method to show effects of the educational context on learning. The setting of an actual learning context is strength of this study and makes the outcomes much more ecologically valid than controlled (lab) experiments, but it also made the study more complex. A pure experimental design, for example, would not have included a control group mixed with a non-randomised group. However, because it was situated in an actual learning context, the study could not avoid mixing the controls with students that do not want to participate or who could not be included. The most important reason was that it would have been unethical to give the non-participants anything but an optimal ‘standard education’. Had we separated out the control group and the non-participants then the participants would have been automatically put in their own small subset and thus be similar to the intervention groups. When analysing their results we found that the GPA was lower in the non-randomised group, which indicates that the latter group consisted of ‘weaker’ students and could affect the students from the control group negatively. No differences were found in group learning processes between the control group and the not randomised student group in the first and second curriculum years. Several interpretations are possible to understand the effects from mixing these two groups on the results and conclusions. First of all, mixing might not have ‘weakened’ the control group nor the non-randomised group. Second, the initially weaker non-randomised group lifted on the ‘stronger’ control group. Finally, the control group leaned on the non-randomised student group and became weaker than would have occurred when not mixed. Although theoretically all three possibilities could have occurred we think that the results suggest that the control group was not weakened nor lifted by the non-randomised groups as no differences in group dynamics were measured from the second week of medical programme and further onwards in the curriculum between the control and non-randomised groups.

Since two intervention groups (A & B) were used in one experiment, each replicating the findings of the other, we conclude that the beneficial effect of the intervention is valid and replicable. The validity of our results are strongly founded on the collaborative learning literature aligning to Kane's notion of an argument-based approach to validity [59]. Since this research was conducted in an actual learning context, we believe that the results are quite unique and underline the ecologic validity of the results, which is normally a critical downside in pure tightly controlled experimental studies.

This study was performed in the context of the Maastricht Medical School, a Problem-Based Learning context. We want to argue that the results of this intervention study are also beneficial to other small-group learning contexts similar to this curriculum design, such as team-based learning contexts. Implementation of this intervention can be rather simple since no additional costs are needed to reducing class size by dividing the class into subsets.

Increasing the strength of the intervention could further accelerate the positive effects. In this study we manipulated only a part of the educational processes (the tutorial groups) and not all other educational activities (practical, lectures). It is plausible to assume that with a stronger intervention even stronger effects would be caused. In a further step-up, for example linking a student group to subgroups of faculty further improvement of the efficacy of formal and informal group learning processes could be achieved, as this study clearly shows that classes need to seem small to reach optimal learning processes. In such study we feel that competency development should be introduced as outcome parameters. Since the foundation of learning in small groups, the quality of learning processes, seems to be positively correlated to ‘smaller classes’, it is of extra interest to study performance of students in a variety of competency domains. This has however not been done in this study since the main parameter that was available in the early curriculum years was medical knowledge. We would value incorporating multiple performance indicators such as communication and collaboration skills in a following study, especially if a stronger intervention has been realized as described in the previous paragraph.

To conclude this study, we advise our medical school and others, which make use of a powerful learning context to change the formal group design and make large classes seem small.

## Supporting Information

Figures S1 **A–D: Learning in small groups in the formal learning context over two curriculum years.** The * represents a significant difference from the control group with a p-value≤0.05. The arrow represents a significant difference when both intervention groups are added to counterbalance the lack of power due to a low number of students in the small subsets in year 2 (circa 40 students). Please note that Y-axis starts at 4 since this was ‘neutral’ on the scale. Figure S1 and Table S1 depict perceptions of the four parameters for effective group processes. These learning processes were assessed in two modules in the first and second curriculum year, observed twice per module. The intervention was expected to take effect when the small groups were randomised to new small groups at least twice. However, to understand and monitor the effects of mixing the control group and the non-randomised group to generate a large subset, we explored what happened in the first two small groups that students were involved in.
(TIF)

Table S1 **Effect sizes of learning in small groups in the formal context two curriculum years.** GLB: Group learning behaviour, Potency: Group Potency, Cohesion: Social cohesion, Safety: Psychological Safety. Effect sizes are given in regression coefficients, with standard errors between brackets. Obs 0: starting point in the module. Slope: increase (β) between the start and the end of the module. C = control group, A & B are the intervention groups (small subsets), and nR is the non-randomised group of students. * signifies p-value≤0.05. Figure S1 and Table S1 depict perceptions of the four parameters for effective group processes. These learning processes were assessed in two modules in the first and second curriculum year, observed twice per module. The intervention was expected to take effect when the small groups were randomised to new small groups at least twice. However, to understand and monitor the effects of mixing the control group and the non-randomised group to generate a large subset, we explored what happened in the first two small groups that students were involved in.
(DOCX)

Film S1 **Students learn in the informal context within the subset that the university assigned the students to.** These learning networks over time are shown in 3D.
(ZIP)
