# Supplementary figures and images for: Medical Students Perceive Better Group Learning Processes when Large Classes Are Made to Seem Small
Source: PLoS One. 2014 Apr 15;9(4):e93328. doi: 10.1371/journal.pone.0093328 (PMC3988014; doi:10.1371/journal.pone.0093328)

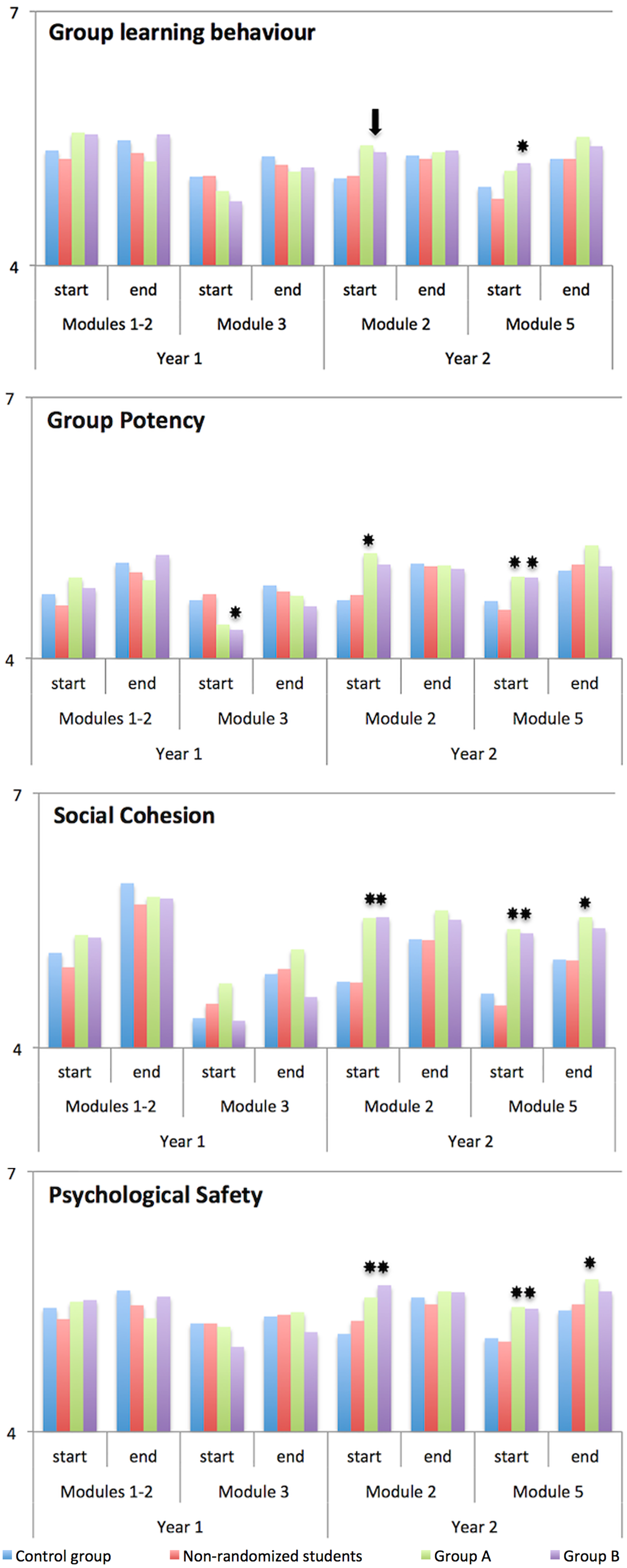

Supplement: Figures S1 — A–D: Learning in small groups in the formal learning context over two curriculum years. The * represents a significant difference from the control group with a p-value≤0.05. The arrow represents a significant difference when both intervention groups are added to counterbalance the lack of power due to a low number of students in the small subsets in year 2 (circa 40 students). Please note that Y-axis starts at 4 since this was ‘neutral’ on the scale. Figure S1 and Table S1 depict perceptions of the four parameters for effective group processes. These learning processes were assessed in two modules in the first and second curriculum year, observed twice per module. The intervention was expected to take effect when the small groups were randomised to new small groups at least twice. However, to understand and monitor the effects of mixing the control group and the non-randomised group to generate a large subset, we explored what happened in the first two small groups that students were involved in. (TIF) [file pone.0093328.s001.tif]
