# Supplementary material for: Medical Students Perceive Better Group Learning Processes when Large Classes Are Made to Seem Small
Source: PLoS One. 2014 Apr 15;9(4):e93328. doi: 10.1371/journal.pone.0093328 (PMC3988014; doi:10.1371/journal.pone.0093328)
Supplement: Table S1 — Effect sizes of learning in small groups in the formal context two curriculum years. GLB: Group learning behaviour, Potency: Group Potency, Cohesion: Social cohesion, Safety: Psychological Safety. Effect sizes are given in regression coefficients, with standard errors between brackets. Obs 0: starting point in the module. Slope: increase (β) between the start and the end of the module. C = control group, A & B are the intervention groups (small subsets), and nR is the non-randomised group of students. * signifies p-value≤0.05. Figure S1 and Table S1 depict perceptions of the four parameters for effective group processes. These learning processes were assessed in two modules in the first and second curriculum year, observed twice per module. The intervention was expected to take effect when the small groups were randomised to new small groups at least twice. However, to understand and monitor the effects of mixing the control group and the non-randomised group to generate a large subset, we explored what happened in the first two small groups that students were involved in. (DOCX) [file pone.0093328.s002.docx]

|  | **GLB** | **Potency** | **Cohesion** | **Safety** |
| --- | --- | --- | --- | --- |
| **Year 1 - Module 1 & 2** | | | | |
| **Obs 0** (C) | *5.34 (.10)* | *4.72 (.10)* | *5.08 (.11)* | *5.40 (.10)* |
| A | 0.25 (.20) | 0.21 (.20) | 0.25 (.20) | 0.12 (.19) |
| B | 0.19 (.20) | 0.08 (.20) | 0.16 (.20) | 0.10 (.10) |
| nR | -0.04 (.10) | -0.08 (.10) | -0.12 (.14) | -0.06 (.10) |
|  |  |  |  |  |
| **SLOPE** (C) | *0.04 (.09)* | *0.29 (.13)** | *0.76 (.12)** | *0.14 (.09)* |
| A | *-0.39 (.16) | -0.31 (.25) | -0.31 (.22) | *-0.34 (.15) |
| B | -0.03 (.16) | 0.10 (.25) | -0.27 (.22) | -0.08 (.15) |
| nR | 0.02 (.12) | 0.11 (.12) | 0.01 (.00) | 0.02 (.12) |
| **Year 1 - Module 3** | | | | |
| **Obs 0** (C) | *5.03* | *4.66 (.08)* | *4.35 (.12)* | *5.25 (.08)* |
| A | -0.15 (.16) | -0.16 (.16) | 0.40 (.23) | -0.03 (.14) |
| B | -0.25 (.16) | *-0.31 (.16) | -0.01 (.23) | -0.25 (.14) |
| nR | 0.02 (.10) | -0.08 (.11) | 0.13 (.13) | -0.02 (.11) |
|  |  |  |  |  |
| **SLOPE** (C) | *0.26 (.07)** | *0.19 (.08)** | *0.54 (.10)** | *0.09 (.08)* |
| A | -0.03 (.12) | 0.03 (.14) | -0.13 (.17) | 0.07 (.13) |
| B | 0.13 (.13) | 0.07 (.14) | -0.27 (.17) | 0.06 (.14) |
| nR | -0.13 (.10) | -0.14 (.11) | -0.12 (.14) | 0.02 (.11) |
| **Year 2 - Module 2** | | | | |
| **Obs 0** (C) | 5.00 (.11) | *4.65 (.12)* | *4.75 (.12)* | *5.10 (.11)* |
| A | 0.42 (.24) | *0.58 (.27) | *0.78 (.24) | *0.47 (.21) |
| B | 0.32 (.24) | 0.42 (.26) | *0.80 (.23) | *0.58 (.21) |
| nR | 0.00 (.11) | 0.04 (.11) | -0.02 (.12) | 0.13 (.12) |
|  |  |  |  |  |
| **SLOPE** (C) | 0.31 (.09)* | 0.44 (.10)* | 0.55 (.10)* | 0.45 (.10)* |
| A | * -0.39 (.15) | *-0.59 (.17) | *-0.46 (.18) | *-0.39 (.17) |
| B | -0.27 (.14) | *-0.48 (.16) | *-0.59 (.17) | *-0.52 (.16) |
| nR | -0.17 (.12) | -0.19 (.13) | -0.11 (.14) | *-0.28 (.13) |
| **Year 2 - Module 5** | | | | |
| **Obs 0** (C) | 4.93 (.08) | *4.66 (.08)* | *4.63 (.09)* | *5.08 (.08)* |
| A | 0.19 (.14) | *0.28 (.13) | *0.77 (.19) | *0.37 (.16) |
| B | * 0.28 (.14) | *0.27 (.13) | *0.73 (.18) | *0.36 (.15) |
| nR | *-0.20 (.10) | -0.06 (.10) | -0.12 (.11) | -0.06 (.11) |
|  |  |  |  |  |
| **SLOPE** (C) | 0.29 (.07)* | 0.32 (.07)* | 0.41 (.08)* | 0.29 (.08)* |
| A | 0.10 (.12) | 0.05 (.13) | *-0.27 (.15) | 0.02 (.14) |
| B | -0.10 (.12) | -0.20 (.13) | *-0.36 (.14) | -0.11 (.13) |
| nR | 0.18 (.10) | 0.11 (.10) | 0.06 (.12) | 0.09 (.11) |
